# Changes in peri-calcarine cortical thickness in blindsight

**DOI:** 10.1016/j.neuropsychologia.2020.107463

**Published:** 2020-06

**Authors:** Loraine Georgy, John D. Lewis, Gleb Bezgin, Matteo Diano, Alessia Celeghin, Alan C. Evans, Marco Tamietto, Alain Ptito

**Affiliations:** a Montreal Neurological Institute, McGill University, Montreal, Canada; b Department of Psychology, University of Torino, Torino, Italy; c Center of Research on Psychology in Somatic Diseases – CoRPS - Tilburg University, the Netherlands; d Department of Psychology, McGill University Health Centre, Montreal, Canada; e McGill Centre for Studies in Aging, Douglas Institute, McGill University, Montreal, Canada

**Keywords:** Cortical thickness, Blindsight, Plasticity, Vision, Hemispherectomy

## Abstract

Blindsight is the ability of patients with primary visual cortex (V1) damage to process information in their clinically blind visual field in the absence of conscious awareness. In addition to those with localized V1 lesions, some patients exhibiting this phenomenon have had a cerebral hemisphere removed or disconnected from the rest of the brain for the treatment of drug-resistant epilepsy (hemispherectomy). Research into the underlying neural substrates of blindsight has long implicated the intact visual cortex in maintaining residual vision and supporting visuo-guided responses to stimuli presented ipsilaterally within the blind visual field while operating outside the geniculo-striate pathway. A recent study demonstrated functional reorganization in the dorsal visual areas of the intact hemisphere, thereby supporting its compensatory role in non-conscious vision. In this study, we used cortical thickness analysis to examine anatomical differences in the visual cortex of the intact hemisphere of three subjects with varying degrees of cortical damage and well documented blindsight: two with a right hemispherectomy (complete and partial), and one with a left V1 lesion. T1-weighted MRI data were obtained for the subjects while control data were chosen from publicly available NKI-dataset to match closely the acquisition parameters of our blindsight cases. Our results show significant increases in cortical thickness in the visual cortex of all blindsight subjects compared to healthy controls, irrespective of age-onset, etiology, and extent of the damage. Our findings add to accumulating evidence from behavioral, functional imaging, and tractography studies of cerebral compensation and reorganization.

## Highlights

• Examined anatomical changes in the intact visual cortex of rare blindsight patients.
• First comparison of hemispherectomy and lesion patients to a large control sample.
• Blindsight subjects show significant increases in peri-calcarine cortical thickness.
• Similar changes observed despite differences in etiology and age at time of lesion.
• Increases are possible morphological signs of compensation underlying blindsight.

## 1 Introduction

Despite damage to their primary visual cortex (V1) which leads to clinical blindness in the corresponding portion of the visual field, some patients are, nevertheless, able to process and respond to stimuli presented in their cortically blind visual field independently of conscious awareness (Pöppel et al., 1973). This phenomenon was termed ‘blindsight’ By Weiskrantz et al. (1974), and research into these functions uncovered the patients' abilities to detect and localize stationary and moving stimuli (Blythe et al., 1987; Stoerig et al., 1985; Zihl and von Cramon, 1980), discriminate stimuli based on motion (Blythe et al., 1986), line orientation (Weiskrantz et al., 1974), colour (Danckert et al., 1998), form (Marcel, 1998), frequency (Magnussen and Mathiesen, 1989), wavelength (Stoerig and Cowey, 1989), categories (Van den Stock et al., 2014), and demonstrate visuo-motor transformation (Celeghin et al., 2017), semantic priming (Marcel, 1998), emotional processing (de Gelder et al., 1999), and navigational skills (de Gelder et al., 2008). Further examination into blindsight has also uncovered evidence of an interaction between stimuli presented simultaneously to the blind and intact visual hemifields (Georgy et al., 2016; Marzi et al., 1986).

This combined evidence points to a potential mechanism for blindsight whereby visual information presented in the presumably blind field utilizes direct projections from subcortical structures to reach extrastriate visual areas bypassing V1 in the undamaged hemisphere (Bridge et al., 2008). Using a variety of imaging techniques, research into the underlying pathways involved in blindsight has implicated a variety of retino-recipient subcortical structures like the superior colliculus (Leh et al., 2010; Tamietto et al., 2010), the pulvinar (Leh et al., 2008), and the lateral geniculate nucleus (Ajina and Bridge, 2018). In addition, research has identified increased anatomical connectivity between lateral geniculate nucleus and motion area MT/V5 (Bridge et al., 2008) not present in patients without blindsight (Ajina et al., 2015), as well as connections between the superior colliculus, pulvinar, and amygdala (Tamietto et al., 2012). In conjunction with evidence of plasticity involving these structures in blindsight, a wealth of functional and structural connectivity studies advocate for the role of the intact hemisphere in compensating altered visual functions (Bittar et al., 1999; Goebel et al., 2001; Leh et al., 2006). Recently, retinotopic mapping performed in a blindsight patient indeed showed functional reorganization of the population receptive field sizes within the dorsal visual areas of the intact hemisphere (Georgy et al., 2019).

One question that remains, however, is whether the functional changes associated with blindsight are accompanied by anatomical modifications in the grey matter microstructure of the visual cortex in the intact hemisphere. While patients with localized V1 lesions have offered a unique perspective on the study of compensation contributed by the intact hemisphere which may be mediated through interhemispheric connections, hemispherectomy patients who have had an entire cerebral hemisphere disconnected anatomically or functionally for the treatment of intractable epilepsy offer an equally unique and rare opportunity to examine the compensatory effects mediated by input from subcortical structures, particularly through the superior colliculus, which is the sole surviving retino-recipient structure in the otherwise damaged hemisphere. We leave the question of identifying the mechanism through which anatomical changes in the visual areas of the intact hemisphere might be occurring to future work; however, as a critical first step towards fully understanding this phenomenon, the present study aims to provide a characterization of these anatomical changes in individuals with varying lesions who, nevertheless, all exhibit blindsight.

Cortical thickness can be estimated from neuroimaging data based on the different magnetic resonance imaging (MRI) signals associated with grey and white matter. It is a brain morphometric measure of the distance between the pial surface and the grey/white matter boundary. This metric has been garnering interest recently for its use in clinical populations to identify morphological changes in the cortex. In the present study we analysed cortical thickness to assess morphological differences in the visual cortex of the intact hemisphere of three subjects with blindsight due to either hemispherectomy or localized V1 damage.

## 2 Materials and methods

### 2.1 Subjects

**Fig. 1:**
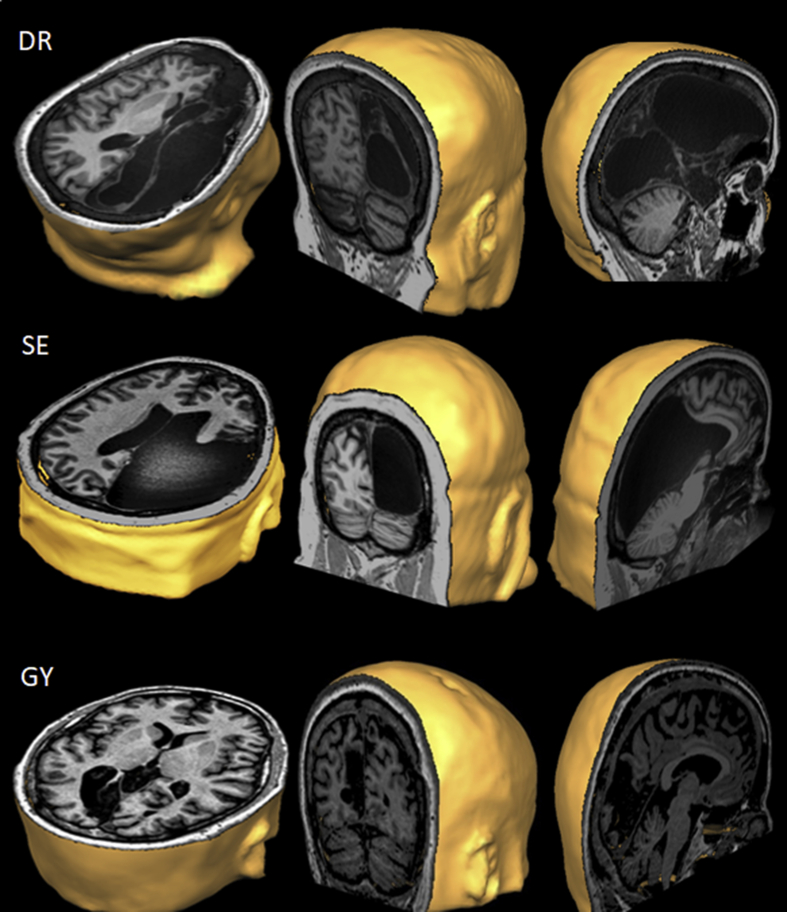
Sections of T1-weighted 3T MRI anatomical scans of DR (top), SE (middle), and GY (bottom) showing the lesion of the visual cortex in transverse (left), coronal (middle) and sagittal (right) views.

We studied three subjects with blindsight: one complete right hemispherectomy patient: DR, one partial right hemispherectomy patient: SE, and one patient with a localized left V1 lesion: GY (Fig. 1), and 188 control subjects.

DR is a right-handed woman (43 years old at the time of testing) with left hemiparesis since birth who began suffering from epileptic seizures at the age of 5. Computerized Tomography (CT) and MRI scans of the brain revealed severe atrophy of the right cerebral hemisphere, and electroencephalography (EEG) studies showed epileptiform activity over the right frontal-parietal-temporal regions. At the age of 17, she underwent a functional hemispherectomy which included removal of the frontal-parietal-temporal lobes including the mesial structures. The remaining cortical regions were disconnected from the rest of the brain by sectioning the white matter anteriorly and laterally, as well as posteriorly and laterally along the falx. Subsequent neuropathological investigation revealed an inflammatory process with diffuse gliosis, characteristic of Rasmussen encephalitis. The presence of a complete contralateral hemianopia without macular sparing was confirmed by computerized perimetry (Allergan, Humphrey), and she has consistently shown strong evidence of blindsight (e.g. Georgy et al., 2016).

SE is a right-handed man (49 years old at the time of testing) whose left hemiparesis was noted at birth with seizure onset at the age of 7. At the age of 23, CT and MRI scans showed a porencephalic cyst occupying the right temporal-parietal-occipital regions. EEG recordings detected epileptiform activity in the right occipital cortex along with independent foci over the right temporo-parietal cortex. At the age of 25, he underwent surgery to remove the congenital porencephalic cyst, and a temporal-parietal-occipital lobectomy including the hippocampus and amygdala sparing the anterior portion of the frontal lobe was performed. Postoperative neuropathological examination revealed a neuronal migration disorder (cortical dysplasia). MRI scans postoperatively and later for research purposes and published elsewhere showed the presence of intact left and right SC, but only an intact pulvinar on the left side (Leh et al., 2008). A contralateral hemianopia without macular sparing was confirmed by computerized perimetry (Allergan, Humphrey), and he has consistently shown strong evidence of blindsight (e.g. Georgy et al., 2016).

GY is a left-handed man (54 years old at the time of testing) who was involved in a traffic accident at the age of 8 causing a vascular incident resulting in a large unilateral lesion in the left medial occipital lobe. The striate cortex is consequently absent in the left hemisphere and the lesion includes the peripheral representation of V1 except at the occipital pole corresponding to 3–4° of macular sparing. There is a smaller lesion in the right parietal lobe that has not been investigated behaviorally. GY's visual system has been highly investigated (Baseler et al., 1999) and he has consistently shown strong evidence of blindsight (e.g. Celeghin et al., 2017).

Control data were chosen from the NKI dataset, a cross-sectional sample of individuals from childhood to senescence; we selected from this set all individuals within 15 years of our youngest and oldest cases. The set of controls which fit this criterion and passed manual quality control is comprised of 188 individuals (mean age = 52.2; standard deviation = 9.5; 140 females; 169 right-handed).

This project was approved by the McGill University Health Centre (MUHC) Research Ethics Board (NEUPSY Panel; NEU-11-026).

### 2.2 MRI acquisition and processing

For all subjects, T1-weighted MR images were acquired using an MPRAGE sequence on a Siemens 3T scanner. DR was scanned on a Tim Trio (Repetition time [TR] = 2300 ms; Echo time [TE] = 2.98 ms; 1 mm isotropic resolution); SE on a Prisma (TR = 2300 ms; TE = 2.98; 1 mm isotropic resolution); GY on an Allegra (TR = 2250 ms; TE 2.6 ms; 1 mm isotropic resolution). All the control subjects were scanned on a Siemens Tim Trio 3T scanner (TR = 1900 ms; TE = 2.52; 1 mm isotropic resolution).

All data were processed with CIVET-2.1.0 (released October 2016; http://www.bic.mni.mcgill.ca/ServicesSoftware/CIVET) to extract cortical thickness measures. CIVET is a fully automated structural image analysis pipeline developed at the Montreal Neurological Institute. Intensity non-uniformities are corrected using N3; the input volume is aligned to the Talairach-like ICBM-152-nl template; the image is classified into white matter, grey matter, cerebrospinal fluid, and background; the white-matter surface is extracted via marching cubes and adjusted to the centre of the gradient at the inner edge of the cortical grey matter; the pial surface is positioned by walking outward from the white-matter surface to the cerebro-spinal fluid; the surfaces are mapped to a common surface template and thickness is measured as the Laplacian distance between the white- and grey-matter surfaces in native space.

**Fig. 2:**
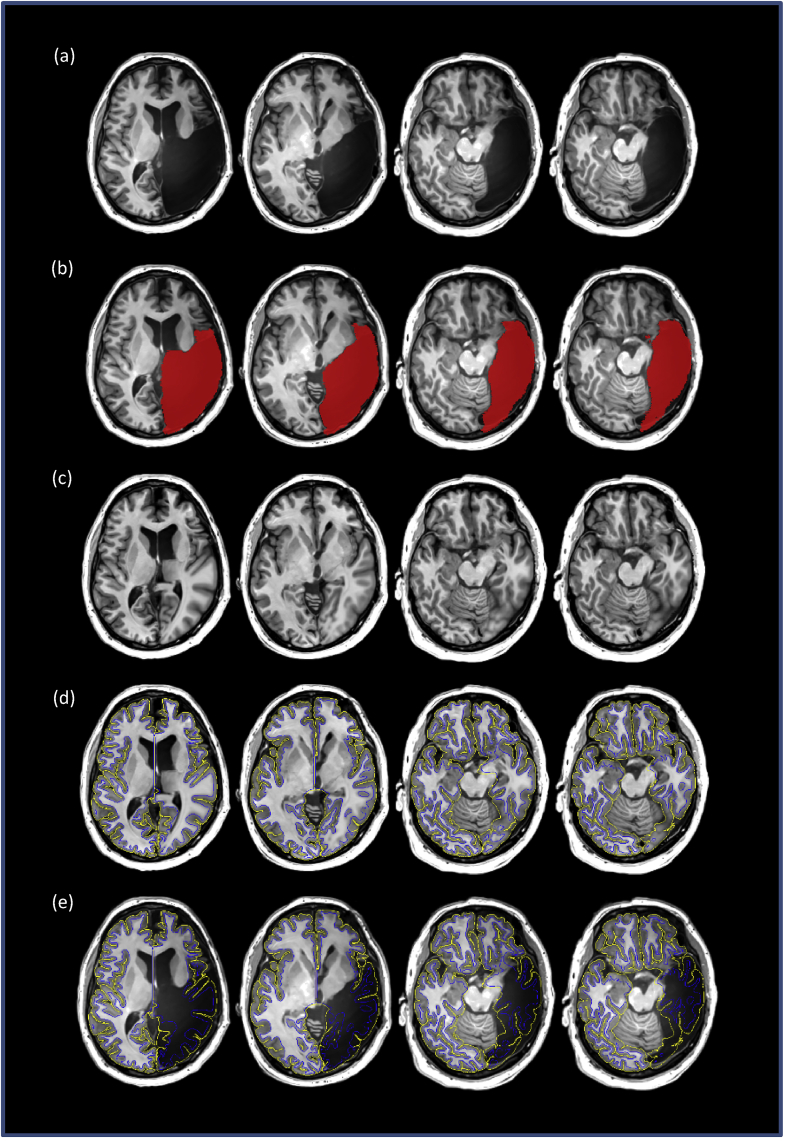
The processing procedure shown on consecutive axial slices for patient SE as an example. The lesioned MRI (a) is manually processed to create a lesion mask (b); the MNI152 template is registered to the lesioned MRI, and a composite brain is constructed by replacing the lesioned area of the brain with its MNI152 counterpart (c). The composite brain is then processed with CIVET to derive the white and grey surfaces (d) which provide measure of CT for the non-lesioned areas of the subject's brain (e).

In order to process the data for patients, their lesions were filled with the corresponding portion of the non-linearly aligned MNI-152 template which was achieved by manually constructing a volumetric mask of the lesion for each subject; this was done using the manual segmentation tools in the MNI Display software (http://www.bic.mni.mcgill.ca/software/Display/Display.html). This lesion mask was then subtracted from the brain mask obtained from the brain extraction tool mincbet. The resulting mask was then used to guide linear and non-linear registration of the MNI-152 template to overlay the subject's MRI, and then the lesion mask was used to fill in the lesioned portion of the subject's brain with the corresponding portion of the MNI-152 template. This composite brain was then processed by CIVET, and cortical thickness values extracted for the non-lesioned portions of the subject's brain. This procedure is illustrated in Fig. 2.

### 2.3 Analysis

Statistical analyses were conducted using the SurfStat statistical toolbox (http://www.math.mcgill.ca/keith/surfstat), implemented in MATLAB. For each of the blindsight cases, a set of coefficients for the linear model

M = 1 + age + sex + age∗sex + handedness

was generated based on the cortical thickness data from all control subjects, with age centered on the age of the particular blindsight case. The coefficients of this model were then used to predict the thickness of the blindsight cases based on their age, sex and handedness (predictedThickness). The studentized residual for the cortical thickness in the peri-calcarine region in the intact hemisphere of the blindsight patients was then calculated as:

studentizedResidual=(actualThickness−predictedThickness)/controlStddevs

where controlStddevs is the standard deviation of the residuals for the control data. The studentized residual was used to identify the set of vertices in each subject that were outliers with respect to the control population. To control for the proportion of Type 1 errors, a random field theory correction for multiple comparisons was performed (Worsley, 2007).

## 3 Results

**Fig. 3:**
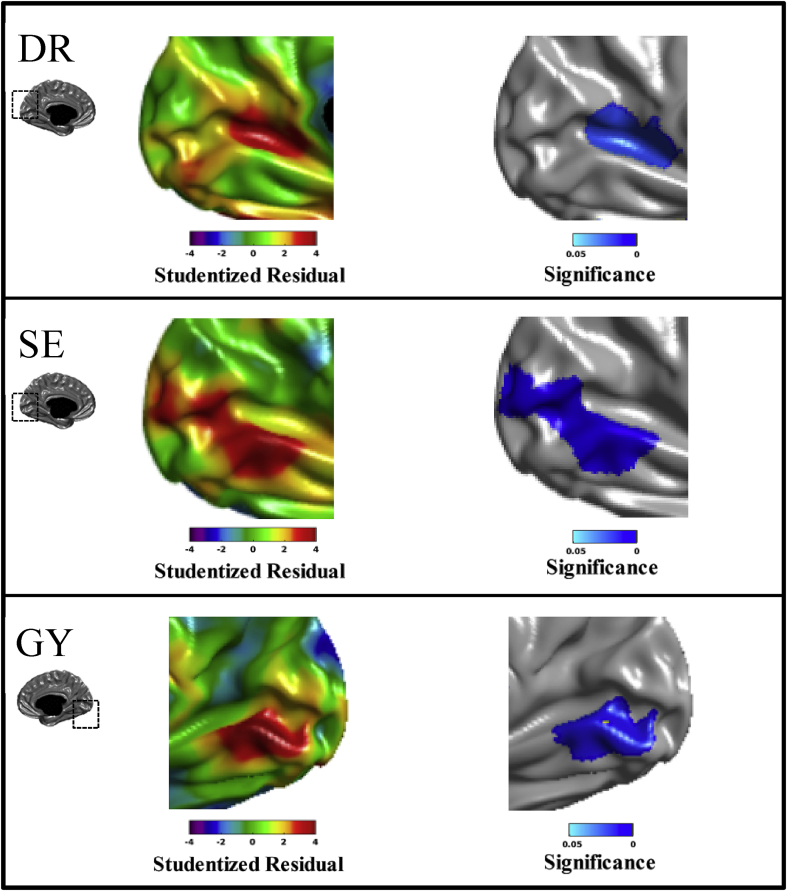
Peri-calcarine cortical thickness abnormalities in blindsight. The studentized residual (left) shows the differences in the CT of the blindsight cases from what is predicted based on healthy controls. The significance maps (right), with multiple comparison correction via random field theory, show significant increases in peri-calcarine regions in each subject. Note that significance maps show cluster-wise significance.

Our results demonstrate significant increases of cortical thickness in the visual cortex of the intact hemisphere in all blindsight cases compared to healthy controls. Fig. 3 shows the studentized residual and the random field theory (RFT) corrected significance maps for each patient. DR shows increased cortical thickness in the anterior part of the calcarine fissure. The mean cortical thickness in this region in DR is 3.05; the predicted cortical thickness in this region for a right-handed 43-year-old female is 2.33. SE shows increased cortical thickness in a more posterior region of the calcarine fissure, extending into the neighbouring peri-striate cortex. The mean cortical thickness in this region in SE is 2.64; the predicted cortical thickness in this region for a right-handed 49-year-old male is 2.02. GY shows increased cortical thickness along the inferior bank of the calcarine fissure extending into the lingual gyrus. The mean cortical thickness in this region in GY is 2.70; the predicted cortical thickness in this region for a left-handed 54-year-old male is 1.94. Note that in all three blindsight cases all regions showing an RFT-corrected significant alteration in cortical thickness include portions of the primary visual cortex.

## 4 Discussion

In this study, we aimed to explore whether there were any measurable changes in cortical thickness in the visual cortex of the intact hemisphere in subjects exhibiting residual vision in their presumed blind field following damage to the corresponding primary visual cortex. In particular, we were interested in the visually responsive areas within the intact hemisphere which were previously shown to be associated with the visual processing pathway in blindsight. We assessed three subjects with blindsight: one who had undergone a complete right hemispherectomy (DR), one who had undergone a partial right hemispherectomy (SE), and one who had a circumscribed left V1 lesion (GY).

There has been a considerable number of findings implicating the intact visual cortex in the processing of visual information presented in the ipsilateral ‘blind’ hemifield in subjects exhibiting blindsight (A. Ptito et al., 2001; M. Ptito et al., 1999). Furthermore, our group previously used functional magnetic resonance imaging (fMRI) to show that visual stimulation of the blind hemifield in DR yielded activation in the ipsilateral intact visual cortex (Bittar et al., 1999), and that some functional reorganization of the receptive field sizes was taking place in these areas (Georgy et al., 2019). In GY, transcranial magnetic stimulation (TMS) applied over the extrastriate areas in his damaged hemisphere was shown to modulate the appearance of phosphenes induced from the intact primary visual cortex, indicating significant interhemispheric functional connectivity (Silvanto et al., 2009).

Our results demonstrate clear anatomical differences in the striate and extrastriate visual cortex within the intact hemisphere of all three subjects compared to healthy controls; there were significant increases in cortical thickness along the calcarine sulcus, which remain consistent across the three patients despite the varying age, nature, cause, and side of their cortical injury. The consistency of this finding, as well as its circumscribed nature, rules out unspecific or general plastic changes, suggesting a relationship between these anatomical differences and the functional role of the intact V1 in mediating residual vision in blindsight.

These differences in cortical thickness together with accumulating evidence from combined behavioral, functional imaging, and tractography studies on the critical role of the intact hemisphere in blindsight following damage to V1 (Celeghin et al. 2015, 2017) provide indications of morphological plasticity within the remaining visual cortex. They lend support to the idea of a dynamic model of reorganization where spared visual functions following destruction of the primary visual cortex can be attributed to the compensatory role of cortical areas in the undamaged hemisphere, feasibly mediated by existing neural pathways from subcortical nuclei.

Before discussing the specific implications of these findings, it is worth considering the interpretation for changes of cortical thickness in the brain. While substantial thinning beyond the developmental epoch of synaptic pruning typically reflects loss or impaired function, implications of cortical thickening are not straightforward. Cortical thickness analyses provide a viable index for brain structure differences but the association of the metric to microstructural changes is unclear. Early studies in animals investigating the microstructure of the brain have shown that increases in cortical thickness are often a result of increased dendritic arborization (e.g. Kolb and Whishaw, 1989) which are reliably reflected in higher synaptic but lower neuronal densities (e.g. Cullen et al., 2010). Imaging and histological work in humans suggest that cortical thickness correlates with increased soma size (Rajkowska et al., 1998) and is inversely related to neuronal density in the occipital cortex (la Fougere et al., 2011). From a behavioral standpoint, human patients with macular degeneration show increased cortical thickness in peripherally-responsive visual areas reflecting compensatory gain of function which leads to spared peripheral vision (Burge et al., 2016). Considering the functional reorganization demonstrated in the intact visual cortex of blindsight subjects, it is reasonable to explain increased cortical thickness as a strengthening of cortico-tectal connections whereby input from remaining subcortical structures, such as the SC, triggers changes in the microstructure of the intact visual cortex in the form of increases in dendritic arborization and synaptic density. Such alterations would enable the processing of visual information from the ipsilateral, cortically blind visual field.

Several studies have used cortical thickness assessment in order to observe neuroplasticity in the human occipital cortex (Elvsashagen et al., 2017) and shown that visual areas and associated cortices are capable of change under a variety of circumstances. For example, Rogge et al. (2018) found increased cortical thickness in the visual and vestibular cortical areas induced by balance-training exercises that rely on extensive vestibulo-visual stimulation. Additionally, this measure was used to investigate cortical changes that accompany brain disorders or appear as a consequence of brain injury. Findings show anatomical differences in the functionally defined visual areas that correlate with visual processing abnormalities in behavioral and neural measures in disorders such as schizophrenia (e.g. Reavis et al., 2017). Cortical thickness within the primary visual cortex has also been heavily studied in blind subjects, showing consistently that changes in visual experience can induce changes in the cortical thickness of V1 (Anurova et al., 2015). Research into these alterations has suggested that congenitally and early blind individuals show a thicker V1 compared to sighted controls (Jiang et al., 2009). There is compelling evidence from the literature suggesting that congenitally or early blind subjects activate their visual cortex in nonvisual tasks (e.g. M. Ptito and Kupers, 2005). In an attempt to relate these functional changes to anatomical changes resulting from sight loss, Voss & Zatorre gathered cortical thickness measures in blind and sighted subjects along with several nonvisual behavioral measures (Voss and Zatorre, 2012). Group contrasts confirmed a thicker occipital cortex in the early blind subjects, which correlated with superior behavioral scores thereby demonstrating a direct link between increased cortical thickness in the visual cortex and adaptive cross-modal reorganization in the brain of the visually deprived (Collignon et al., 2009; Pascual-Leone et al., 2005; cf. Singh et al., 2018). While these findings support the capacity of the visual cortex to functionally and anatomically reorganize in order to support plastic changes in sensory perception, and are therefore important to discuss in relation to our findings, it is necessary to contextualize the important distinction between the visual cortex of the congenitally blind and that in the remaining hemisphere of blindsight subjects. In the visually deprived, the brain makes use of cortical tissue that receives virtually no visual input, and possibly reorganizes crossmodally, both functionally and anatomically, to support nonvisual tasks. By contrast, the remaining visual cortex in blindsight subjects remains intact, and continues to support normal vision contralaterally. In that regard, the blindsight pathway is thought to use these existing visual networks that remain intact in order to support vision following contralateral V1 damage.

However, the loss of interhemispheric connections between the striate cortices could provide an alternate explanation for our results. It is possible that increases in cortical thickness in the intact visual cortex of our hemispherectomy subjects are associated with a decrease in invading myelinated fibers that would normally connect the visual cortices. In this way, such a loss may displace the surface at the inner edge of the cortex further inward. It is worth noting, however, that we do see the same effect of cortical thickening in our patient with a localized V1 lesion who shows no damage to the posterior corpus callosum and preserved intact interhemispheric connections between the undamaged visual areas surrounding the lesion and the contralateral hemisphere (Celeghin et al., 2017). Still, we cannot exclude the possibility that the loss of V1 in the damaged hemisphere leads to an increase in cortical thickness that is not functionally driven; this question would require further histological work in hemispherectomy and lesion patients post mortem.

It is important to note that while our study shows anatomical changes that persist in three patients who have little in common outside total unilateral destruction of V1 and well documented blindsight, it is not possible to unequivocally attribute these increases in cortical thickness to either the damage and subsequent compensatory changes, or to the underlying functional reorganization that mediates blindsight. Indeed, the phenomenon of cortical reorganization as a means of sensory compensation remains highly debated (Singh et al., 2018). It is, therefore, important to compare the findings reported here to cortical thickness changes in the visual cortex of the intact hemisphere in hemispherectomy and/or lesion patients who do not demonstrate evidence of blindsight. Future research should also examine changes in cortical thickness in other visually responsive areas, particularly those that mediate visuo-motor response such as the temporal visual areas, and posterior parietal and frontal premotor areas.

## Author contribution statement

Dr. Loraine Georgy is the first author of the article and took a lead role in the preparation of the study titled *“Changes in peri-calcarine cortical thickness in blindsight”*. Dr. Marco Tamietto was involved in conceiving the experiment with Dr. Alain Ptito and Dr. Loraine Georgy. Two of the subjects were recruited by Dr. Alain Ptito and one subject was recruited by Dr. Marco Tamietto. Dr. Alessia Celeghin and Dr. Matteo Diano assisted Dr. Loraine Georgy in data collection through a collaborative effort. Dr. Gleb Bezgin acquired the control data from the NKI database; Dr. John D. Lewis procesed the data for cortical thickness analysis; and Dr. Loraine Georgy performed quality control measures. Dr. Loraine Georgy and Dr. John D. Lewis collaborated on the data analysis. All authors were involved in the interpretation of results and preparation of the manuscript.

## CRediT authorship contribution statement

**Loraine Georgy:** Conceptualization, Data curation, Methodology, Formal analysis, Validation, Visualization, Writing - original draft, Writing - review & editing. **John D. Lewis:** Data curation, Methodology, Formal analysis, Validation, Visualization, Writing - original draft, Writing - review & editing. **Gleb Bezgin:** Data curation, Writing - review & editing. **Matteo Diano:** Data curation, Visualization, Writing - review & editing. **Alessia Celeghin:** Data curation, Writing - review & editing. **Alan C. Evans:** Funding acquisition, Supervision, Writing - review & editing. **Marco Tamietto:** Conceptualization, Funding acquisition, Supervision, Data curation, Writing - review & editing. **Alain Ptito:** Conceptualization, Funding acquisition, Supervision, Data curation, Writing - review & editing.
